# Risk Factors and Outcomes of *Stenotrophomonas maltophilia* Bacteraemia: A Comparison with Bacteraemia Caused by *Pseudomonas aeruginosa* and *Acinetobacter* Species

**DOI:** 10.1371/journal.pone.0112208

**Published:** 2014-11-06

**Authors:** Go Hotta, Yasufumi Matsumura, Karin Kato, Satoshi Nakano, Tomoyuki Yunoki, Masaki Yamamoto, Miki Nagao, Yutaka Ito, Shunji Takakura, Satoshi Ichiyama

**Affiliations:** 1 Kyoto University Graduate School of Medicine, Department of Clinical Laboratory Medicine, 54 Shogoin-Kawaharacho, Sakyo-ku, Kyoto, Japan; 2 Kyoto University Graduate School of Medicine, Department of Respiratory Medicine, 54 Shogoin-Kawaharacho, Sakyo-ku, Kyoto, Japan; Animal Health and Veterinary Laboratories Agency, United Kingdom

## Abstract

*Stenotrophomonas maltophilia* (SM) is an important nosocomial pathogen that exhibits intrinsic resistance to various antimicrobial agents. However, the risk factors for SM bacteraemia have not been sufficiently evaluated. From January 2005 to September 2012, we retrospectively compared the clinical backgrounds and outcomes of SM bacteraemic patients (SM group) with those of bacteraemic patients due to *Pseudomonas aeruginosa* (PA group) or *Acinetobacter* species (AC group). DNA genotyping of the SM isolates using the Diversilab system was performed to investigate the genetic relationships among the isolates. The SM, PA, and AC groups included 54, 167, and 69 patients, respectively. Nine of 17 patients in the SM group receiving trimethoprim-sulfamethoxazole prophylaxis developed SM bacteraemia. Independent risk factors for SM bacteraemia were the use of carbapenems and antipseudomonal cephalosporins and SM isolation within 30 days prior to the onset of bacteraemia. Earlier SM isolation was observed in 32 of 48 patients (66.7%) with SM bacteraemia who underwent clinical microbiological examinations. Of these 32 patients, 15 patients (46.9%) had the same focus of bacteraemia as was found in the previous isolation site. The 30-day all-cause mortality rate among the SM group (33.3%) was higher than that of the PA group (21.5%, p = 0.080) and the AC group (17.3%, p = 0.041). The independent factor that was associated with 30-day mortality was the SOFA score. DNA genotyping of SM isolates and epidemiological data suggested that no outbreak had occurred. SM bacteraemia was associated with high mortality and should be considered in patients with recent use of broad-spectrum antibiotics or in patients with recent isolation of the organism.

## Introduction

*Stenotrophomonas maltophilia* (SM) is an emerging nosocomial pathogen. In a surveillance performed from 1997 to 1999 in the Asia-Pacific, Europe, Latin America, Canada, and the United States regions, SM was the third most frequently isolated non-fermentative gram-negative bacilli, following *Pseudomonas aeruginosa* (PA) and *Acinetobacter* species (AC) [1]. SM is intrinsically resistant to beta-lactams or aminoglycosides via the chromosomal resistance genes L1 and L2 beta-lactamase and aminoglycoside-modifying enzymes [2]. Trimethoprim-sulfamethoxazole (TMP-SMZ) is the first-line therapeutic drug for the treatment of SM, and it is recommended that severe infections be treated with a high dose of TMP-SMZ, similar to the dose used to treat *Pneumocystis jirovecii* [2]. Thus, the therapeutic options for SM are quite different from those available for other non-fermentative gram-negative bacilli, and the appropriate antimicrobial therapy is often delayed [3]. Therefore, a high crude mortality rate among SM bacteraemic patients has been reported, ranging from 14% to 69% [4]. Several studies have evaluated the risk factors for SM bacteraemia, but these studies have involved small populations or patients with specific medical conditions, such as haematological malignancies [5]–[9]. No study has compared SM bacteraemia in all hospitalised patients with bacteraemias due to PA or AC, which are the most important nosocomial pathogens. To elucidate the clinical characteristics of SM bacteraemia, we compared the antimicrobial susceptibility, clinical backgrounds, and prognostic factors of SM bacteraemic patients with those of patients suffering from bacteraemia due to *Pseudomonas aeruginosa* (PA) or *Acinetobacter* species (AC). The risk factors for 30-day mortality were evaluated in the SM bacteraemic patients. For the SM isolates, DNA genotyping was also conducted to investigate the genetic relationships among the SM isolates.

## Methods

### Ethics statement

The Ethics Committee of Kyoto University Graduate School of Medicine (E-2070) approved this study and waived the need for obtaining informed consent from each patient.

### Setting and study design

This study was conducted at Kyoto University Hospital, an 1121-bed tertiary-care hospital in Kyoto, Japan. All of the bacteraemic episodes in the hospital were reported to and were followed up by our infectious disease physicians. From January 2005 to September 2012, all patients who had positive blood cultures for SM, PA, or AC were enrolled. Patients who had blood cultures that were positive for more than one bacterial species of SM, PA, and AC were excluded. Each patient was included in the study only once, at the time of the initial blood culture. A case control-control study design was used. The cases consisted of patients with SM bacteraemia (SM group), and the first and second control groups was consisted of patients with PA bacteraemia (PA group), or patients with AC bacteraemia (AC group), respectively.

### Variables and definitions

The clinical information acquired from the medical charts included age, sex, duration of hospital stay, presence of polymicrobial infection, underlying comorbidities, Charlson score [10], Sequential Organ Failure Assessment (SOFA) score [11], presence of septic shock [12], history of organ transplantation, surgery within 30 days, neutropenia, administration of immunosuppressive drugs, use of mechanical support or indwelling catheters, ICU stay and duration, isolation of SM within 30 days, clinical specimen types from which SM was isolated, focus of infection, empirical antimicrobial therapy, and 30-day mortality rate. Polymicrobial infection was defined as the identification of two or more bacterial species in blood culture samples collected within 72 hours. Nosocomial bacteraemia was defined as bacteraemia that occurred 72 hours or more after admission. Neutropenia was defined as an absolute neutrophil count less than 500/µl at the onset of bacteraemia. The administration of immunosuppressive drugs included corticosteroids or other immunosuppressive drugs within 14 days before the onset of bacteraemia. Previous antimicrobial therapy was defined as the administration of antibiotics for more than 48 hours within 14 days prior to the onset of bacteraemia. The isolation of SM within 30 days was defined as the isolation of SM from an extra-blood site between 1 and 30 days prior to the onset of bacteraemia. The focus of infection was clinically determined based on an active infection site and on the isolation of the organism from the site coincident with the onset of bacteraemia. Empiric antimicrobial therapy was considered to be inappropriate if an active antimicrobial agent, as determined by *in vitro* susceptibility testing, was not administered during the first 72 hours after the blood sample was obtained. The attributable mortality (bacteraemia-related deaths) was judged by our two infectious diseases physicians when the patient would not have died in the absence of bacteraemia.

To evaluate the risk factors for the 30-day all-cause mortality rate of the SM group, the clinical background, severity of illness, and rate of appropriate therapy among the patients who did not survive were compared with those of the patients who survived.

### Antimicrobial susceptibilities

Blood culture samples were processed using the BACTEC 9240 system (Becton Dickinson Microbiology Systems, Sparks, MD, USA). All positive cultures were gram stained and subcultured on blood agar plates and bromothymol blue (BTB) agar plates for further identification. An automatic identification system, the Vitek2 system (bioMéreux, Marcy l’Etoile, France), and the Micro Scan WalkAway (Siemens Healthcare Diagnostics, Tokyo, Japan) were used to identify SM, PA, and AC. The antimicrobial susceptibilities were evaluated using the broth microdilution method, and were categorised according to the 2012 Clinical Laboratory Standard Institute (CLSI) breakpoints [13]. For agents without published CLSI criteria for SM, the relevant criteria for non-Enterobacteriaceae were used [13]. To test the susceptibilities of SM and AC to tigecycline, an Etest (bioMéreux, Marcy l’Etoile, France) was performed using Iso-Sensitest agar [14].

### DNA genotyping

The automated rep-PCR Diversilab Microbial Typing System (Sysmex-bioMéreux, Marcy l’Etoile, France) was used to investigate the clonal relationship of the SM isolates, according to manufacturer’s recommendations. The resulting analysis was performed using the Diversilab software (version 3.4), which uses the Pearson correlation coefficient to determine the distance matrices and the unweighted pair group method with arithmetic averages to create dendrograms. Isolates with a similarity of at least 95% were considered a cluster.

### Statistical analysis

The categorical variables were compared using the Chi squared test or Fisher’s exact test. The continuous variables were compared using the Mann-Whitney U test. To determine the independent risk factors for SM bacteraemia and for the 30-day all-cause mortality, all of the variables with a p-value of <0.05 based on univariate analyses were subjected to further selection using a forward stepwise logistic regression. The survival curves for the patients with SM, PA, and AC bacteraemia were prepared according to the Kaplan-Meier method. The log-rank test was used to compare the survival curves. A p-value of <0.05 was considered to be statically significant. We conducted our statistical analyses with Stata version 11.2 (StataCorp, College Station, TX, USA).

## Results

We identified 54 patients with SM bacteraemia, 167 patients with PA bacteraemia, and 69 patients with AC bacteraemia. No patients with bacteraemia were positive for more than one bacterial species.

Table 1 lists the results of the univariate analysis of the clinical characteristics of the SM, PA, and AC groups. All of the SM patients, 82.0% of the PA patients, and 89.9% of the AC patients had nosocomial bacteraemia. Seventeen patients in the SM group had received TMP-SMZ within 14 days of the onset of bacteraemia for prophylaxis against *Pneumocystis jirovecii* pneumonia (trimethoprim 80 mg daily). Breakthrough infection (bacteraemia during TMP-SMZ prophylaxis) was observed in 9 patients, and 4 patients developed TMP-SMZ resistant SM bacteraemia. When compared with the PA and AC groups, the SM patients were characterised by a longer hospital stay, receiving intensive care, an indwelling catheter, previous antimicrobial therapy, and the isolation of SM within 30 days. Urinary tract infections were not observed in the SM group, whereas these infections were found in 14.4% of the PA patients. An elevated SOFA score, solid organ transplantation, previous treatment with aminoglycosides, and respiratory infectionwere significantly more common in the SM group than in the AC group. Multivariate analysis with each control (the PA and the AC groups) revealed that the same factors were independently associated with SM bacteraemia, including previous treatment with carbapenems or antipseudomonal cephalosporins and isolation of SM within 30 days (Table 2).

**Table 1:** Clinical characteristics of *S. maltophilia* bacteraemic patients compared to *P. aeruginosa* and *Acinetobacter* species bacteraemic patients: univariate analysis.

Clinical backgrounds	SM (N = 54)	PA (N = 167)	AC (N = 69)	SM vs. PA	SM vs. AC
	no. (%)	no. (%)	no. (%)	p-value	p-value
Age, median (IQR)	56 (39.8–65.3)	61 (49–70)	62 (37.5–72)	0.033	0.133
Sex	26 (48.1)	99 (59.3)	39 (56.5)	0.151	0.356
Underlying comorbidities					
Solid malignancy	21 (38.9)	52 (31.1)	23 (33.3)	0.292	0.524
Haematological malignancy	7 (13.0)	35 (21.0)	8 (11.6)	0.193	0.818
Diabetes	12 (22.2)	39 (23.4)	12 (17.4)	0.864	0.502
Renal dysfunction	9 (16.7)	33 (19.8)	13 (18.8)	0.614	0.755
Heart diseases	8 (14.8)	16 (9.6)	8 (11.6)	0.283	0.598
Liver diseases	19 (35.2)	47 (28.1)	14 (20.3)	0.326	0.064
Respiratory diseases	3 (5.6)	14 (8.4)	6 (8.7)	0.769	0.730
Autoimmune diseases	9 (16.7)	22 (13.2)	10 (14.5)	0.521	0.741
Charlson score, median (IQR)	3 (2–5)	3 (2–5)	3 (2–4)	0.858	0.218
Medical condition					
Nosocomial bacteraemia^a^	54 (100.0)	137 (82.0)	62 (89.9)	<0.001	0.018
Duration of hospital stay, median (IQR)	50 (28–95)	27 (13–52)	29 (13–56)	<0.001	0.002
SOFA score, median (IQR)	6 (2–10)	4 (2–8)	3 (1–6)	0.070	0.002
Solid organ transplantation	17 (31.5)^b^	45 (26.9)	10 (14.5)	0.519	0.024
Bone marrow transplantation	5 (9.3)	11 (6.6)	2 (2.9)	0.548	0.238
Operation within previous 30 days	17 (31.5)	37 (22.2)	14 (20.3)	0.166	0.156
Mechanical ventilation	22 (40.7)	25 (15.0)	7 (10.1)	<0.001	<0.001
CRRT	10 (18.5)	8 (4.7)	1 (1.4)	<0.001	0.001
Maintenance-haemodialysis	3 (5.6)	8 (4.8)	4 (5.8)	0.732	1.000
Neutropenia	8 (14.8)	41 (24.6)	4 (5.8)	0.134	0.128
Central venous catheter	36 (66.7)	81 (48.5)	29 (42.0)	0.020	0.005
Urethral catheter	34 (63.0)	64 (38.3)	25 (36.2)	0.002	0.003
Nasogastric tube	29 (53.7)	43 (25.7)	22 (31.9)	<0.001	0.015
Drainage tube	31 (57.4)	52 (31.1)	26 (37.7)	0.001	0.029
Immunosuppressive agents	29 (53.7)	95 (56.9)	29 (42.0)	0.682	0.076
ICU admission	19 (35.2)	16 (9.6)	7 (10.1)	<0.001	0.001
Previous antimicrobial therapy					
Carbapenems	22 (40.7)	24 (24.0)	10 (14.5)	<0.001	0.001
Glycopeptides	29 (53.7)^c^	32 (19.2)	18 (26.1)	<0.001	0.002
Antipseudomonal cephalosporins	30 (55.6)	34 (20.4)	12 (17.4)	<0.001	<0.001
Non-antipseudomonal cephalosporins	8 (14.8)	23 (13.8)	10 (14.5)	0.848	0.960
Antipseudomonal penicillins	6 (11.1)	15 (9.0)	5 (7.2)	0.643	0.533
Non-antipseudomonal penicillins	4 (7.4)	17 (10.2)	5 (7.2)	0.790	1.000
Fluoroquinolones	9 (16.7)	16 (9.6)	7 (10.1)	0.153	0.289
Aminoglycosides	5 (9.3)	8 (4.8)	0 (0.0)	0.314	0.015
TMP-SMZ	17 d (31.5)	61 (36.5)	19 (27.5)	0.500	0.633
Minocycline	4 (7.4)	1 (0.6)	1 (1.4)	0.013	0.168
SM isolation within 30 days	32 (66.7^e^)	10 (8.3^e^)	4 (8.3^e^)	<0.001	<0.001
Site of infection					
Respiratory	8 (14.8)	20 (11.9)	2 (2.9)	0.586	0.019
Catheter-related	12 (22.2)	21 (12.5)	15 (21.7)	0.080	0.949
Intra-abdominal	12 (22.2)	25 (15.0)	7 (10.1)	0.215	0.066
Urinary tract	0 (0.0)	24 (14.4)	4 (5.8)	0.002	0.130
Skin and soft tissue	0 (0.0)	9 (5.4)	2 (2.9)	0.117	0.503
Primary	22 (40.7)	68 (40.7)	38 (55.1)	0.998	0.082
Inappropriate empiric therapy	17 (31.5)	9 (5.4)	4 (5.8)	<0.001	<0.001
30-day mortality					
All-cause mortality	18 (33.3)	36 (21.6)	12 (17.4)	0.080	0.041
Attributable mortality	12 (22.2)	27 (16.2)	7 (10.1)	0.310	0.066

SM, *S. maltophilia*; PA, *P. aeruginosa*; AC, *Acinetobacter* species; IQR, interquartile range; SOFA, Sequential Organ Failure Assessment; CRRT, continuous renal replacement therapy; ICU, Intensive care unit; TMP-SMZ, trimethoprim-sulfamethoxazole.

a All of the patients other than those with nosocomial bacteraemia had underlying comorbidities and had been followed up in the outpatient department.

b Antipseudomonal cephalosporins or carbapenems were administered in 13 patients (76.4%).

c Antipseudomonal cephalosporins or carbapenems were administered in 26 patients (89.6%).

d All of these patients received TMP-SMZ for prophylaxis against *Pneumocystis jirovecii* pneumonia.

e Microbiological examinations were performed in 48 patients (88.9%) in the SM group, in 120 patients (71.9%) in the PA group, and in 48 patients (69.6%) in the AC group. The percentages in the Table represent the number of patients with SM isolation divided by the number of patients who underwent microbiological examination.

**Table 2:** Risk factors of *S. maltophilia* bacteraemia compared to the bacteremias due to *P. aeruginosa* and *Acinetobacter* species: multivariate analysis.

Clinical backgrounds	SM vs. PA		SM vs. AC	
	OR (95%CI)	p-value	OR (95%CI)	p-value
Use of carbapenems	2.8 (1.1–6.8)	<0.001	3.0 (1.0–9.0)	0.047
Use of antipseudomonal cephalosporins	4.0 (1.8–9.0)	0.001	4.1 (1.5–11.2)	0.005
Isolation of SM within 30 days	16.4 (6.7–39.6)	0.019	12.0 (3.5–40.3)	<0.001

SM, *S. maltophilia*; PA, *P. aeruginosa*; AC, *Acinetobacter* species; OR, odds ratio; CI, confidence interval.

Forty-eight patients in the SM group (88.9%) underwent microbiological examinations within 30 days prior to the onset of bacteraemia as a part of routine clinical practice. The number of specimens obtained from each patient ranged 3 to 64 (median: 14.5). Thirty-two patients (66.7%) had previously been positive for SM. The median duration between the first isolation of SM and the onset of bacteraemia was 11 days (interquartile range: 3–20 days). The most frequent site of previous isolation of SM were lower respiratory tract (96.8%, 30 of 31 examined patients), followed by biliary tract (53.8%, 7 of 13 patients), peritoneal cavity (27.3%, 6 of 22 patients), and central venous catheter tip (17.4%, 4 of 23 patients). Table 3 demonstrates the association between the previous SM isolation site and the focus of SM bacteraemia. Fifteen patients (46.9%) were considered as having secondary bacteraemia from the previous isolation sites, and 10 patients (31.3%) were considered as having primary bacteraemia. Among the 7 patients considered as bacteraemia from another site of infection, 6 had catheter related infections and 1 patient had a biliary tract infection.

**Table 3:** Previous *S. maltophilia* isolation site among the 32 *S. maltophilia* bacteraemic patients and the corresponding focus of bacteraemia.

Focus of bacteraemia	Site of *S. maltophilia* isolation within 30 days prior tothe onset of bacteraemia, no. (%)				
	Respiratory tract(N = 30)	Biliary tract^a^(N = 7)	Peritoneal cavity(N = 6)	Cental venouscatheter tip (N = 4)	Overall (N = 32)
Identical to the previous isolationsite (secondary bacteraemia)	8 (26.7)	3 (42.9)	2 (33.3)	2 (50.0)	15 (46.9)
Other site	13^b^ (43.3)	3^c^ (42.9)	3^d^ (50.0)	1^e^ (25.0)	7^f^ (21.9)
Primary bacteraemia	9 (30.0)	1 (13.7)	1 (16.7)	1 (25.0)	10 (31.3)

a All of the specimens were obtained from drainage tubes.

b Catheter-related, n = 8; biliary tract, n = 3; peritoneal cavity, n = 2.

c Respiratory tract, n = 1; peritoneal cavity, n = 1; catheter-related, n = 1.

d Respiratory tract, n = 1; biliary tract, n = 1; catheter-related, n = 1.

e Peritoneal cavity.

f Catheter-related, n = 6; biliary tract, n = 1.

**Figure 1:**
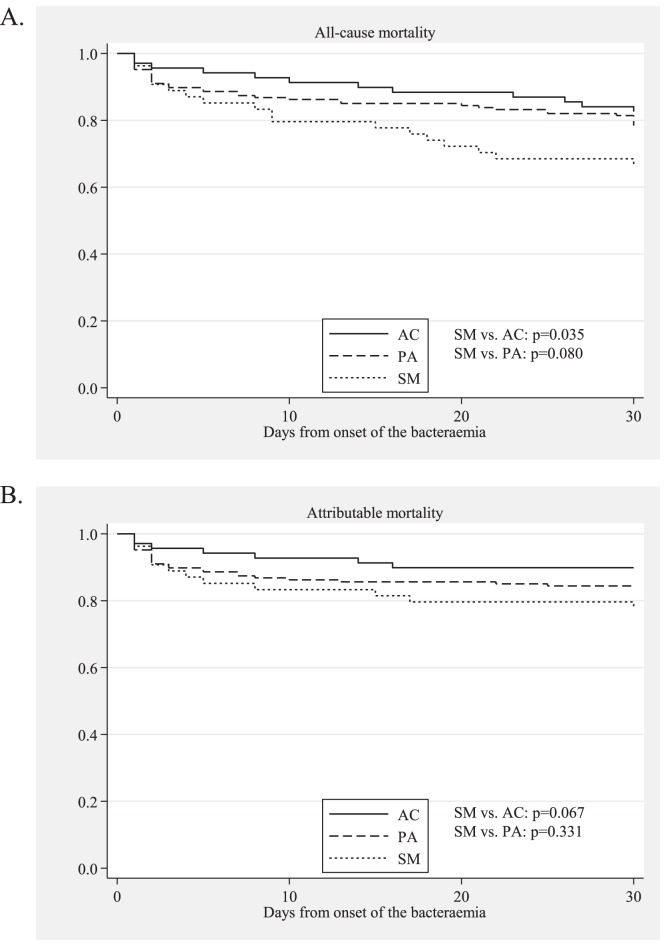
Kaplan-Meyer survival curves for patients with bacteraemia caused by *S. maltophilia* (SM), *P. aeruginosa* (PA), and *Acinetobacter* species (AC). Panel A shows the all-cause mortality, and panel B shows the attributable mortality. The p-values were calculated using the log-rank test. The median times and interquartile ranges to death among the SM, PA, and AC patients were 8.5 (2–18), 5 (2–21.5), and 12 (3.5–24.5) days for the all-cause mortality and 3.5 (2–11.5), 2 (1–7), and 5 (1–14) days for the attributable mortality, respectively.

The SM group received inappropriate antimicrobial therapy more frequently than the PA or the AC group (p<0.001 for each). The 30-day all-cause and attributable mortality rates among the SM group (33.3% and 22.2%, respectively) were higher than the rates in the PA and AC groups, although a significant difference was only observed in the comparison of the all-cause mortality of the AC group (17.4%, p = 0.041). Fig. 1 shows the Kaplan-Meier survival curves for the SM, PA, and AC groups.

The risk factors for the 30-day all-cause mortality in the SM group, according to the univariate analysis, are shown in Table 4. An elevated Charlson or SOFA score, septic shock, ICU stay, mechanical ventilation, continuous renal replacement therapy, and the presence of a urethral catheter or drainage tube were associated with the 30-day mortality of SM patients. Catheter-related infections were associated with survival. Inappropriate antimicrobial therapy was not associated with a poor prognosis. The SOFA score was independently associated with 30-day mortality (OR: 1.3; 95% CI: 1.1–1.5) according to the multivariate analysis.

**Table 4:** Risk factors for 30-day all-cause mortality among the *S. maltophilia* bacteraemic patients: univariate analysis.

Factors	Non-survivors (N = 18)	Survivors (N = 36)	OR (95%CI)	p-value
	no. (%)	no. (%)		
Sex (male)	7 (36.8)	19 (52.8)	0.6 (0.2–0.8)	0.336
Age, median (IQR)	51.5 (42–62.3)	57.5 (39.3–66.8)		0.640
Duration of hospital stay, median (IQR)	51 (28.3–100.3)	46.5 (28–101.8)		0.993
Polymicrobial infection	5 (27.8)	8 (22.2)	1.3 (0.4–4.9)	0.448
Underlying diseases				
Solid malignancy	7 (38.9)	14 (38.9)	1.0 (0.3–3.2)	0.619
Haematological malignancy	1 (5.6)	6 (16.7)	0.3 (0.03–2.7)	0.403
Diabetes	4 (22.2)	8 (22.2)	1.0 (0.2–3.9)	1.000
Renal dysfunction	5 (27.8)	4 (11.1)	3.1 (0.7–13.3)	0.142
Heart disease	4 (22.2)	4 (11.1)	2.3 (0.4–10.5)	0.418
Liver disease	7 (38.9)	12 (33.3)	1.3 (0.4–4.1)	0.456
Lung disease	1 (5.6)	2 (5.6)	1.0 (0.08–11.8)	1.000
Autoimmune disease	4 (22.2)	5 (13.9)	1.8 (0.4–7.6)	0.461
Charlson score, median (IQR)	4 (3–6)	2.5 (2–4)		0.033
Medical condition				
SOFA score, median (IQR)	13.5 (7–14.3)	4 (2–7)		<0.001
Septic shock	11 (61.1)	5 (13.9)	9.7 (2.6–37.1)	<0.001
Solid organ transplantation	8 (44.4)	9 (25.0)	2.4 (0.7–7.9)	0.147
Bone marrow transplantation	1 (5.6)	4 (11.1)	0.5 (0.04–4.5)	0.655
Surgery within 30 days	8 (44.4)	9 (25.0)	2.4 (0.7–7.9)	0.147
Neutropenia	2 (11.1)	6 (16.7)	0.6 (0.1–3.5)	0.704
ICU stay	12 (66.7)	7 (19.4)	8.3 (2.3–29.8)	0.001
Immunosuppressive agents	12 (66.7)	17 (47.2)	2.2 (0.7–7.3)	0.177
Mechanical ventilation	13 (72.2)	9 (25.0)	7.8 (2.1–28.0)	<0.001
Maintenance-haemodialysis	0 (0.0)	3 (8.3)	0.3 (0.01–5.3)	0.543
CRRT	7 (38.9)	3 (8.3)	11.0 (2.4–49.3)	0.011
Central venous catheter	15 (83.3)	21 (58.3)	3.5 (0.9–14.5)	0.066
Urethral catheter	15 (83.3)	19 (52.8)	4.5 (1.1–18.1)	0.027
Nasogastric tube	12 (66.7)	17 (47.2)	2.2 (0.7–7.3)	0.177
Drainage tube	14 (77.8)	17 (47.2)	3.9 (1.1–14.2)	0.032
SM isolation within 30 days^a^	12 (70.6)	20 (64.5)	1.3 (0.3–4.8)	0.757
Site of infection				
Respiratory^b^	5 (27.8)	3 (8.3)	4.2 (0.9–20.3)	0.100
Catheter-related^b^	1 (5.6)	11 (30.6)	0.1 (0.2–1.0)	0.044
Intra-abdominal	3 (16.7)	9 (25.0)	0.6 (0.1–2.6)	0.730
Primary	9 (50.0)	13 (36.1)	1.7 (0.5–5.6)	0.386
Inappropriate empiric therapy	6 (33.3)	11 (30.6)	1.1 (0.3–3.8)	0.836

OR, odds ratio; CI, confidence interval; IQR, interquartile range; SOFA, Sequential Organ Failure Assessment; ICU, Intensive care unit; CRRT, continuous renal replacement therapy.

a Microbiological examinations were performed in 17 non-survivors (94.4%) and in 31 survivors (94.4%).

b Patients with respiratory tract infections had a significantly higher risk for mortality than patients with catheter-related infections (OR, 18.3; 95% CI, 1.5–223; p = 0.018).

Table 5 shows the antimicrobial susceptibilities of the three groups. The SM isolates had high susceptibility rates to minocycline (100%), tigecycline (94.4%), TMP-SMZ (81.5%), and levofloxacin (79.6%).

**Table 5:** Antimicrobial susceptibilities of the blood isolates of *S. maltophilia*, *P. aeruginosa,* and *Acinetobacter* species.

Antibiotics	SM (N = 54), %	PA (N = 167), %	AC (N = 69), %
Amikacin	11.1^a^	96.4	97.1
Levofloxacin	79.6	76.9^b^	95.7
Meropenem	0.0^a^	76.6	95.7
Ceftazidime	42.6^a^	92.9	85.5
Cefepime	3.7^a^	88.5	88.4
TMP-SMZ	81.5	ND	91.2
Minocycline	100.0	ND	98.6
Tigecycline	94.4	ND	98.6

TMP-SMZ, trimethoprim-sulfamethoxazole; SM, *S. maltophilia*; PA, *P. aeruginosa*; AC, *Acinetobacter* species; ND, not done.

a The susceptibility rate was significantly lower compared with PA or AC isolates.

b Analysis of the susceptibility was performed for the 157 available isolates.

**Figure 2:**
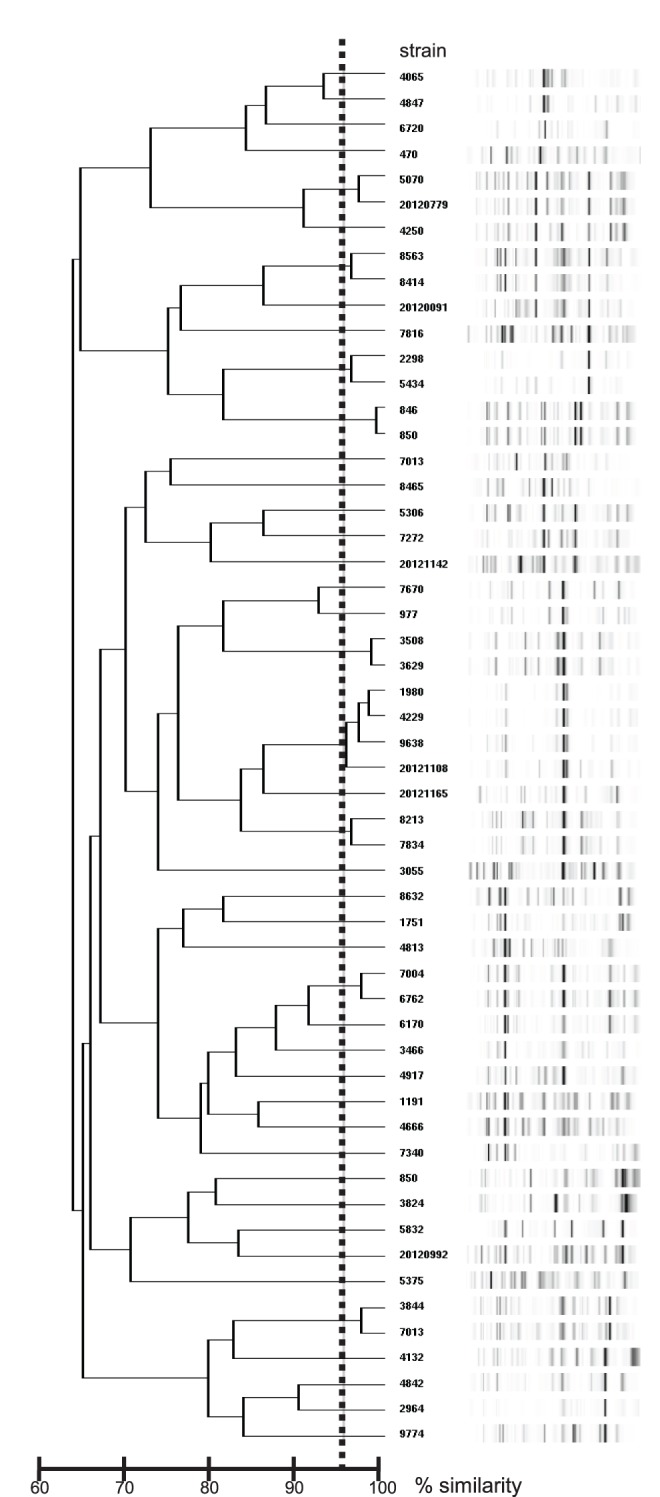
Genotyping of the 54 *S. maltophilia* isolates using the Diversilab system. Twenty isolates belonged to nine clusters. One cluster contained four isolates, whereas the other eight clusters contained two isolates each.

No apparent outbreak of SM bacteraemia was observed during the study period. DNA genotyping using the Diversilab system of the 54 SM isolates showed that 20 isolates belonged to the same cluster. One cluster contained four isolates, whereas the other eight clusters contained two isolates each (Fig. 2). The isolates that belonged to each cluster were not epidemiologically related; the related strains were detected in distant wards, or there was a period of greater than six months between the detection of the related strains.

## Discussion

This is the first study that investigated the risk factors for SM bacteraemia in all hospitalised patients, in comparison with other major non-fermentating gram negative bacilli. Independent risk factors found to be associated with SM bacteraemia included the use of carbapenems and antipseudomonal cephalosporins and the isolation of SM within 30 days. The SOFA score was an independent prognostic factor.

Many studies have reported that the majority of the SM patients have haematological malignancies, with a rate ranging from 29% to 57% [5], [7], [15]–[17]. In our study, SM patients commonly had solid malignancies (38.9%) and chronic liver diseases (35.2%), or underwent solid organ transplantation (31.5%); patients with haematological malignancies (13.0%) or bone marrow transplant recipients (9.3%) were less common. Greater than 60 patients per year underwent liver transplantation in this hospital. Solid organ transplant recipients undergo surgical procedures and they are exposed to mechanical ventilation, haemodialysis, drainage or vascular catheter insertion, and broad-spectrum antimicrobials for a long period of time. These features might be one reason why our SM bacteraemia cases were associated with solid organ transplant recipients more than with bone marrow transplant recipients.

The use of carbapenems and antipseudomonal cephalosporins were the independent risk factors for SM bacteraemia. The risk factors for SM bacteraemia when compared with *Escherichia coli* bacteraemic or non-bacteraemic patients have included the use of carbapenems, glycopeptides, aminoglycosides, central venous catheter [5], [7]. The use of cefepime or other antipseudomonal drugs including aminoglycosides has been described as the risk factors for SM infection in patients with critically ill trauma or cystic fibrosis [18], [19]. In univariate analysis with the AC group, the use of aminoglycosides was also the risk factor. SM is generally considered as resistant to antipseudomonal beta-lactams and aminoglycosides. Therefore, it is presumed that use of these agents may predispose to SM colonization or infection. The use of glycopeptides was also the risk factor for SM in univariate analysis, but this was not significant in multivariate analysis. The SM patients who were previously treated with glycopeptides were frequently treated with carbapenems or antipseudomonal cephalosporins. The previous use of prophylactic TMP-SMZ was not a negative risk factor for SM bacteraemia, and breakthrough SM bacteraemia occurred in approximately half of the patients and was frequently associated with TMP-SMZ resistance. Low dose treatment with TMP-SMZ might allow breakthrough infection and predispose the patient to resistant bacteraemia.

Another independent risk factor for SM bacteraemia was isolation of SM within 30 days. No previous study had indicated that the isolation of SM was an independent risk factor for SM bacteraemia. Nseir et al. [20] found that 80% of patients infected with SM had prior isolation of SM, although the association between colonization and infection was not evaluated. In case of *A. baumanii* bacteraemia, one case-control study identified that colonization with *A. baumanii* was the most significant risk factor [21]. The previous isolation site might be of value for predicting the source of bacteraemia because approximately half of the patients had secondary bacteraemia. The majority of cases of bacteraemia from sites other than the previous isolation site were central venous catheter-related cases. This result might be associated with the fact that a specimen from a vascular catheter tip could not be easily obtained and is often removed after presence of bacteraemia.

Compared to both control groups, mechanical support, intensive care, and indwelling catheters were significant risk factors for SM bacteraemia, although these factors were not independent. Mechanical ventilation and ICU stay has been shown as risk factors of SM infection [22], [23]. SM adheres to the abiotic surfaces of medical implants or indwelling catheters and an association between colonization of SM and prosthetic devices has been observed [24]. Therefore, these devices may play a role in the occurrence of SM bacteraemia. Urinary tract infections were more frequently observed in the PA group than in the SM group. Well-documented cases of SM urinary tract infections are rare [25], [26], whereas PA often causes urinary tract infections in a hospital setting [27]. A history of solid organ transplantation was more frequently observed among the SM group than the AC group. The SM patients who received solid organ transplantation prior to bacteraemia were frequently received carbapenems and antipseudomonal cephalosporins.

In the univariate analysis, SM bacteraemic patients with catheter-related infections had better outcomes compared with other sites of infection or respiratory tract infections; these results are consistent with the results of previous studies [28], [29]. The severity of the illness was related to the poor outcome of SM bacteraemia, and administration of the inappropriate antimicrobial treatment was not associated with increased mortality. The impact of early empirical treatment on SM infection remains to be elucidated [30]. Some studies suggest that bacteraemia due to SM was directly influenced by the conditions of each patient [15], [16], [26], [28], [31]. However, other studies have reported that the initial administration of the inappropriate antibacterial treatment to patients was a significant predictor of mortality [5], [17], [18], [32]–[33].

Antimicrobial therapy for SM infection is problematic because many isolates are resistant to multiple agents used to treat gram-negative infections. In our study, isolates of the SM group showed antimicrobial susceptibilities that were distinct from those of the PA and the AC groups, and the SM group frequently received inappropriate empiric antimicrobial therapy. Similar to previous reports, the SM isolates in this study were resistant to beta-lactams and aminoglycosides. The resistance of SM to TMP-SMZ is problematic when treating SM infection. The resistance rates are reported to be between 8% and 18% in the Asia-Pacific, and the SM isolates in the present study showed a similar resistance rate [1], [34]. Levofloxacin may be an alternative drug to treat SM infection [35]. Recent studies indicated that levofloxacin was not inferior to TMP-SMZ for the treatment of SM infection or bacteraemia [36], [37]. However, rapid resistance to fluoroquinolones has been observed *in vitro* and *in vivo* [2]. Minocycline, which was active against all of the SM isolates, may also be used to treat SM infection, although its clinical application is still limited [2].

DNA genotyping using the Diversilab system revealed that the majority (63%) of the SM isolates were genetically unrelated. Among the genetically related isolates, no epidemiological linkage was observed, and these results suggest that there were no outbreaks during the study period. The SM clinical isolates also showed high genodiversity in previous reports [5], [38].

The present study has several limitations that should be acknowledged. First, it was retrospectively performed at a single institution. Second, although this is the largest study to investigate the risk factors of SM bacteraemia, the number of patients in the SM group may still be too small to analyse the prognostic factors of SM bacteraemia. Third, although prior SM isolation was the independent risk factor for SM bacteraemia, we could not perform an active surveillance culture for SM. Active surveillance cultures to predict the occurrence of drug-resistant gram-negative bacteraemia have been reported as a useful method for guiding empiric therapy in critically ill patients [39]. Eighty-nine percent of our patients underwent at least 3 bacterial cultures, and SM was recovered from as many as 66.7% of patients. We believe that this finding justifies further research evaluating the effect of active surveillance for SM.

In conclusion, SM bacteraemia was associated with longer hospital stay, higher mortality and inappropriate empiric antimicrobial therapy compared to bacteraemia due to other major non-fermentative, gram-negative bacilli. In our study, the use of carbapenems and antipseudomonal cephalosporins in the 14 days prior to bacteraemia and the isolation of SM within 30 days were significant risk factors for the development of SM bacteraemia. Furthermore, the judicious use of antipseudomonal beta-lactams is needed, as our study suggests that these agents are a risk factor for developing SM bacteraemia.

## References

[1] Gales AC, Jones RN, Forward KR, Liñares J, Sader HS, et al (2001) Emerging importance of multidrug-resistant *Acinetobacter* species and *Stenotrophomonas maltophilia* as pathogens in seriously ill patients: geographic patterns, epidemiological features, and trends in the SENTRY Antimicrobial Surveillance Program (1997–1999). Clin Infect Dis 32: S104–113. 11320451 10.1086/320183

[2] Looney WJ, Narita M, Mühlemann K (2009) *Stenotrophomonas maltophilia*: an emerging opportunist human pathogen. Lancet Infect Dis 9: 312–323. 19393961 10.1016/S1473-3099(09)70083-0

[3] Fihman V, Le Monnier A, Corvec S, Jaureguy F, Tankovic J, et al (2012) *Stenotrophomonas maltophilia*: The most worrisome threat among unusual non-fermentative gram-negative bacilli from hospitalized patients: A prospective multicenter study. J Infect 64: 391–398. 22245400 10.1016/j.jinf.2012.01.001

[4] Brooke S (2012) *Stenotrophomonas maltophilia*: an emerging global opportunistic pathogen. Clin Microbiol Rev 25: 2–41. 22232370 10.1128/CMR.00019-11 PMC3255966

[5] Senol E, DesJardin J, Stark PC, Barefoot L, Snydman DR (2002) Attributable mortality of *Stenotrophomonas maltophilia* bacteremia. Clin Infect Dis 34: 1653–1656. 12032905 10.1086/340707

[6] Micozzi A, Venditti M, Monaco M, Friedrich A, Taglietti F, et al (2000) Bacteremia due to *Stenotrophomonas maltophilia* in patients with hematologic malignancies. Clin Infect Dis 31: 705–711. 11017819 10.1086/314043

[7] Metan G, Hayran M, Hascelik G, Uzun O (2006) Which patient is a candidate for empiric therapy against *Stenotrophomonas maltophilia* bacteraemia? An analysis of associated risk factors in a tertiary care hospital. Scand J Infect Dis 38: 527–531. 16798705 10.1080/00365540500452481

[8] Apisarnthanarak A, Mayfield JL, Garison T, McLendon PM, DiPersio JF, et al (2003) Risk factors for *Stenotrohphomonas maltophilia* bacteremia in oncology patients: A case-control study. Infect Control Hosp Epidermiol 24: 269–274. 10.1086/502197 12725356

[9] Victor MA, Arpi M, Bruun B, Jønsson V, Hansen MM (1994) *Xanthomonas maltophilia* bacteraemia in immunocompromised haematological patients. Scand J Infect Dis 26: 163–170. 8036472 10.3109/00365549409011780

[10] Charlson ME, Pompei P, Ales KL, MacKenzie CR (1987) A new method of classifying prognostic comorbidity in longitudinal studies: development and validation. J Chron Dis 40: 373–383. 3558716 10.1016/0021-9681(87)90171-8

[11] Ferreira FL, Bota DP, Bross A, Mélot C, Vincent JL (2001) Serial evaluation of the SOFA score to predict outcome in critically ill patients. JAMA 286: 1754–1758. 11594901 10.1001/jama.286.14.1754

[12] Bone RC, Balk RA, Cerra FB, Dellinger RP, Fein AM, et al (1992) Definitions for sepsis and organ failure and guidelines for the use of innovative therapies in sepsis. The ACCP/SCCM Consensus Conference Committee. American College of Chest Physicians/Society of Critical Care Medicine. Chest 101: 1644–1655. 1303622 10.1378/chest.101.6.1644

[13] Clinical and Laboratory Standards Institute (2012) Performance standards for antimicrobial susceptibility testing; twenty-second informational supplement M100-S22. CLSI, Wayne, PA, USA.

[14] Cohen Stuart J, Mouton JW, Diederen BM, Al Naiemi N, Thijsen S, et al (2010) Evaluation of Etest to determine tigecycline MICs for *Enterobacter* species. Antimicrob Agents Chemother 54: 2746–2747. 20350941 10.1128/AAC.01726-09 PMC2876410

[15] Muder RR, Harris AP, Muller S, Edmond M, Chow JW, et al (1996) Bacteremia due to *Stenotrophomonas* (*Xanthomonas*) *maltophilia*: a prospective, multicenter Study of 91 episodes. Clin Infect Dis 22: 508–512. 8852971 10.1093/clinids/22.3.508

[16] Araoka H, Baba M, Yoneyama A (2010) Risk factors for mortality among patients with *Stenotrophomonas maltophilia* bacteremia in Tokyo, Japan, 1996–2009. Eur J Clin Microbiol Infect Dis 29: 605–608. 20177726 10.1007/s10096-010-0882-6

[17] Friedman ND, Korman TM, Fairley CK, Franklin JC, Spelman DW (2002) Bacteraemia due to *Stenotrophomonas maltophilia*: an analysis of 45 episodes. J Infect 45: 47–53. 12217732 10.1053/jinf.2002.0978

[18] Hanes SD, Demirkan K, Tolley E, Boucher BA, Croce MA, et al (2002) Risk factors for late-onset nosocomial pneumonia caused by *Stenotrophomonas maltophilia* in critically ill trauma patients. Clin Infect Dis 35: 228–235. 12115086 10.1086/341022

[19] Denton M, Todd NJ, Littlewood JM (1996) Role of anti-pseudomonal antibiotics in the emergence of *Stenotrophomonas maltophilia* in cystic fibrosis patients. Eur J Clin Microbiol Infect Dis 15: 402–405. 8793400 10.1007/BF01690098

[20] Nseir S, Di Pompeo C, Brisson H, Dewavrin F, Tissier S, et al (2006) Intensive care unit-aquired *Stenotrophomonas maltophilia*: incidence, risk factors, and outcome. Crit Care 10: R143. 17026755 10.1186/cc5063 PMC1751051

[21] Jang TN, Lee SH, Huang CH, Lee CL, Chen CY (2009) Risk factors and impact of nosocomial *Acinetobacter baumanii* bloodstream infections in the adult intensive care unit: a case-control study. J Hosp Infect 73: 143–150. 19716203 10.1016/j.jhin.2009.06.007

[22] Trouillet JL, Chastre J, Vuagnat A, Jolly-Guillou ML, Combaux D, et al (1998) Ventilator-associated pneumonia caused by potencially drug-resistant bacteria. Am J Respir Crit Care Med 157: 531–539. 9476869 10.1164/ajrccm.157.2.9705064

[23] Marshall WF, Keating MR, Anhalt JP, Steckelberg JM (1989) *Xanthomonas maltophilia*: an emerging nosocomial pathogen. Mayo Clin Proc 64: 1097–1104. 2682049 10.1016/s0025-6196(12)64979-9

[24] de Oliveira-Garcia D, Dall'Agnol M, Rosales M, Azzuz AC, Martinez MB, et al (2002) Characterization of flagella produced by clinical strains of *Stenotrophomonas maltophilia.* . Emerg Infect Dis 8: 918–923. 12194767 10.3201/eid0809.010535 PMC2732543

[25] Vartivarian SE, Papadakis KA, Anaissie EJ (1996) *Stenotrophomonas (Xanthomonas) maltophilia* urinary tract infection: a disease that is usually severe and complicated. Arch Intern Med 156: 433–435. 8607729

[26] Samonis G, Karageorgopoulos DE, Makari S, Levis P, Dimopoulou D, et al (2012) *Stenotrophomonas maltophilia* infections in a general hospital: patient characteristics, antimicrobial susceptibility, and treatment outcome. PLoS One 7: e37375. 22624022 10.1371/journal.pone.0037375 PMC3356252

[27] Horcajada JP, Shaw E, Padilla B, Pintado V, Calbo E, et al (2012) Healthcare-associated, community-acquired and hospital-acquired bacteraemic urinary tract infections in hospitalized patients: a prospective multicentre cohort study in the era of antimicrobial resistance. Clin Microbiol Infect 19: 962–968. 23279375 10.1111/1469-0691.12089

[28] Lai CH, Chi CY, Chen HP, Chen TL, Lai CJ, et al (2004) Clinical characteristics and prognostic factors of patients with *Stenotrophomonas maltophilia* bacteremia. J Microbiol Immunol Infect 37: 350–358. 15599467

[29] Boktour M, Hanna H, Ansari S, Bahna B, Hachem R, et al (2006) Central venous catheter and *Stenotrophomonas maltophilia* bacteremia in cancer patients. Cancer 106: 1967–1973. 16565968 10.1002/cncr.21846

[30] Paez JI, Costa SF (2008) Risk factors associated with mortality of infections caused by *Stenotrophomonas maltophilia*: a systematic review. J Hosp Infect 70: 101–108. 18621440 10.1016/j.jhin.2008.05.020

[31] Garazi M, Singer C, Tai J, Ginocchio CC (2012) Bloodstream infections caused by *Stenotrophomonas maltophilia*: a seven-year review. J Hosp Infect 81: 114–118. 22494851 10.1016/j.jhin.2012.02.008

[32] Tunger O, Vural S, Cetin CB, Keles G, Gazi H (2007) Clinical aspects and risk factors of nosocomial *Stenotrophomonas maltophilia* bacteremia episodes in a Turkish intensive care unit. J Chemother 19: 658–664. 18230546 10.1179/joc.2007.19.6.658

[33] Metan G, Uzun O (2005) Impact of initial antimicrobial therapy in patients with bloodstream infections caused by *Stenotrophomonas maltophilia* . Antimicrob Agents Chemother 49: 3980–3981. 16127088 10.1128/AAC.49.9.3980-3981.2005 PMC1195400

[34] Wu H, Wang JT, Shiau YR, Wang HY, Lauderdale TL, et al (2012) A multicenter surveillance of antimicrobial resistance on *Stenotrophomonas maltophilia* in Taiwan. J Microbiol Immuno Infect 45: 120–126. 10.1016/j.jmii.2011.09.028 22154599

[35] Nicodemo AC, Paez JI (2007) Antimicrobial therapy for *Stenotrophomonas maltophilia* infections. Eur J Clin Microbiol Infect Dis 26: 229–237. 17334747 10.1007/s10096-007-0279-3

[36] Wang YL, Scripione MR, Dubrovskava Y, Papadopoulos J (2014) Monotherapy with fluoroquinolone or trimethoprim-sulfamethoxazole for treatment of *Stenotrophomonas maltophilia* infections. Antimicrob Agents Chemother 58: 176–82. 24145530 10.1128/AAC.01324-13 PMC3910778

[37] Cho SY, Kang CI, Kim J, Ha YE, Chung DR, et al (2014) Can levofloxacin be a useful alternative to trimethoprim-sulfamethoxazole for treating *Stenotrophomonas maltophilia* bacteremia? Antimicrob Agents Chemother 58: 581–3. 24126583 10.1128/AAC.01682-13 PMC3910801

[38] Wu ZB, Pan SM, Yu J, Liu Y, Yang XF, et al (2011) Use of Diversilab repetitive sequence-based polymerase chain reaction system for genotyping *Stenotrophomonas maltophilia* isolates in different wards of one hospital. J Hosp Infect 79: 172–188. 21802174 10.1016/j.jhin.2011.06.006

[39] Baba H, Nimmo GR, Allworth AM, Boots RJ, Hayashi Y, et al (2011) The role of surveillance cultures in the prediction of susceptibility patterns of gram-negative bacilli in the intensive care unit. Eur J Clin Microbiol Infect Dis 30: 739–44. 21222134 10.1007/s10096-010-1146-1

